# Thermo-Mechanical Characterization of GFRP Molded Grating Composites Exposed to Elevated Temperatures

**DOI:** 10.3390/polym18141722

**Published:** 2026-07-13

**Authors:** Emrah Madenci, Muhammed İhsan Özgün, Ceyhun Aksoylu, Yasin Onuralp Özkılıç

**Affiliations:** 1 Department of Civil Engineering, Necmettin Erbakan University, 42090 Konya, Türkiye; 2 Department of Technical Sciences, Western Caspian University, Baku 1001, Azerbaijan; 3 Department of Metallurgy and Material Engineering, Necmettin Erbakan University, 42090 Konya, Türkiye; 4 Department of Civil Engineering, Konya Technical University, 42250 Konya, Türkiye; 5 Department of Unique Buildings and Constructions Engineering, Don State Technical University, Gagarin Sq. 1, 344003 Rostov-on-Don, Russia

**Keywords:** GFRP gratings, thermo-mechanical behavior, elevated temperature exposure, fiber–matrix degradation, flexural strength

## Abstract

This study comprehensively investigates the thermal and mechanical degradation behavior of molded glass-fiber-reinforced plastic (GFRP) grating composites subjected to temperatures ranging from 80 °C to 320 °C. Three types of industrially produced GFRP gratings—open-type (OG), thin closed-skin (CG), and thick closed-skin (TCG)—were evaluated using mechanical, microstructural, chemical, and crystallographic analyses. Three-point bending tests revealed that TCG-type specimens exhibited superior thermal resistance, experiencing only a 43.9% loss in strength at 320 °C, whereas OG-type specimens showed significant resin degradation, fiber–matrix decomposition, and microcrack formation at temperatures above 200 °C. Scanning Electron Microscopy (SEM) and Fourier Transform Infrared Spectroscopy (FTIR) analyses revealed significant resin degradation, fiber–matrix decomposition, and microcrack formation. Thermogravimetric analysis (TGA) and Differential Scanning Calorimetry (DSC) confirmed substantial mass loss and structural disintegration at temperatures above 200 °C. Dynamic Mechanical Analysis (DMA) results revealed that the glass transition temperature (Tg) occurred at approximately 115–120 °C. The second-order regression model developed to estimate flexural strength under increasing temperature provided high accuracy (R^2^ > 0.99) for all grating types. It should be noted that this investigation focuses on the short-term thermo-mechanical response under fundamental flexural loading to provide an accurate baseline for preliminary engineering design. The findings emphasize that the effect of temperature should be considered a critical parameter in the structural design of GFRP systems, especially in industrial environments with temperatures above 120 °C. Accordingly, tables for material selection and load-carrying capacity should be recalibrated to account for short-term temperature effects.

## 1. Introduction

Glass-fiber-reinforced polymer (GFRP) composite systems are among the alternative building materials that have become increasingly widespread in construction and infrastructure engineering applications in recent years. Thanks to advantages such as high specific strength, low density, superior corrosion resistance, electromagnetic impermeability and low maintenance requirements, GFRPs offer longer-lasting and sustainable solutions compared to traditional materials such as steel and concrete, especially in applications such as platforms, bridges, facilities and walkways where aggressive environmental conditions prevail [1,2,3,4,5].

Molded GFRP gratings, a type of composite structural system, are rigid grating systems that can carry biaxial loads and are produced by molding continuous glass fibers in unsaturated polyester or vinyl ester-based resin systems [6,7]. These structures predominantly have square or rectangular cell geometries and are preferred for many application areas, such as industrial floor coverings, platform systems, stair steps, and drainage top covers, because of their mechanical and chemical resistance. However, the mechanical performance of GFRP materials is highly sensitive to environmental conditions, especially temperature changes. This situation constitutes a factor that can seriously affect the safety of grid systems used in platforms that operate under direct sunlight, beneath hot process lines, or in industrial facilities with hot air currents, especially during summer months. In many studies on the mechanical behavior of GFRP gratings, properties such as flexural strength, modulus of elasticity, and residual strength have been investigated [8]. Shokrieh and Heidari-Rarani [9] modeled the theoretical flexural behavior of gratings; Izzuddin and Akbar [10] revealed the effect of different binder resins (vinyl ester, polyester, phenolic) on the strength. Gattesco et al. [6] analyzed the effects of boundary conditions and cover plates with both experimental and numerical modeling. On the other hand, studies such as Hosseini et al. [11] investigated the residual behavior of these structures after impact. However, most of these studies were conducted at room temperature, and the temperature parameter was not included in the experimental design. Moreover, these studies considered only resin-based parameters and did not analyze the causal relationships between the integrated structural and microstructural deterioration and flexural behavior.

When the glass transition temperature (Tg) of resin-based matrices is approached, undesirable effects such as matrix softening, bond ruptures at the fiber–matrix interface, internal stress increases, microcrack formation and macroscopic strength loss occur in the material [12,13,14,15]. The glass transition temperature of a polymer is the important parameter that must be considered in design as it limits maximum service temperature at which the structure’s performance is preserved [16,17]. Under exposure to elevated temperature, significant reductions in mechanical properties (tensile strength, modulus of elasticity, etc.) may be encountered [18]. Therefore, the fire-resistant design of GFRP is critical for service safety. Bai and Keller [19] conducted a tensile test on pultruded GFRP laminates with a fiber–matrix ratio of 61 wt % at various elevated temperatures ranging from ambient temperature to 220 °C. The tensile strength at 220 °C was only about 20% of that at ambient temperature. Under thermal loads, the mechanical behavior of sandwich structures having GFRP facings and PET foam core was studied by Rezaei et al. [20]. They have experienced moderate reductions in tensile and compressive strengths up to 175 °C. Similarly, Mazzuca et al. [21] evaluated the fire resistance of vacuum-infused GFRP sandwich panels with PET and PUR cores, demonstrating that panel architecture and passive fire insulation significantly delay thermal collapse. Jafari et al. [22] carried out tensile testing on GFRP laminates differing in fiber orientation in an elevated temperature regime between room temperature and 550 °C. It was determined that approximately 40% of their capacity remained in the unidirectional laminates under extreme temperature conditions.

Comparative and systematic experimental studies conducted on GFRP grating specimens subjected to varying temperatures are scarce in the literature. Most existing sources focus on either connector type or structural form factor, while the interaction between temperature and mechanical behavior is largely ignored. This deficiency creates significant uncertainty regarding safe, long-term use of GFRP systems designed for real field conditions subject to temperature effects.

In this study, the mechanical, microstructural, chemical, and thermal behavior of GFRP composite grids under high-temperature conditions was systematically investigated. In the experimental studies, GFRP grids produced by the molding method were used, and temperature treatments of 80 °C, 120 °C, 200 °C, 240 °C, 280 °C, and 320 °C were applied to these grids. The aim is to evaluate the effects of increasing temperatures on the performance of these structural elements using a multifaceted approach. The findings obtained through three-point bending tests, thermogravimetric analysis (TGA), dynamic mechanical analysis (DMA), Fourier transform infrared spectroscopy (FTIR), X-ray diffraction (XRD), and scanning electron microscopy (SEM) analyses conducted in this study show that GFRP gratings weaken significantly, both structurally and chemically, as temperature increases. At temperatures of 200 °C and above, fiber–matrix interface separations, crack formation in the resin matrix, and deterioration of chemical bonds were clearly observed. The bending strength decreased rapidly when the glass transition temperature was exceeded; the rigidity and ductility of the structure were lost. The results indicate that, to ensure the safe use of molded GFRP gratings in structural engineering applications, certain limits must be defined based on the operating temperature. At temperatures above 120 °C, existing load-carrying tables may be inadequate. Therefore, load-deformation relationships should be recalibrated to account for temperature effects; the ambient temperature in which the gratings will be used should be considered a critical parameter in material selection during the design process. In addition, grating geometry, support points, and installation methods should be reviewed for applications exposed to high temperatures.

### Novelty and Objectives of the Study

This study is one of the first comprehensive investigations in the literature to examine the thermomechanical properties of molded GFRP (Glass Fiber Reinforced Polymer) floor gratings subjected to temperatures ranging from 80 °C to 320 °C, using a multifaceted approach. While factors such as flexural strength, post-impact strength, and resin type have been addressed in the literature from various perspectives, the holistic evaluation of microstructural, chemical, and crystallographic deterioration due to temperature has largely been neglected. In this context, the originality of this study stems from the simultaneous evaluation, using interdisciplinary methods, of parameters such as residual flexural strength, glass transition temperature, mass loss, functional group degradation, and crystalline-amorphous equilibrium changes following controlled-temperature procedures applied to three GFRP grating types (OG, CG, TCG).

Crucially, unlike conventional uniform flat laminates or pultruded sections widely documented in the literature, industrially molded gratings exhibit a complex, monolithic cellular structure with intersecting web walls. The scientific novelty of this study lies in uncovering how macro-geometric boundaries—namely open-cell configurations versus thin and thick integrated secondary top-skin closures—modulate the microstructural degradation rates of the core composite material. This framework introduces the concept of geometry-driven passive thermal shielding (‘skin effect’), in which the outer protective configurations function as physical sacrificial barriers that undergo advanced carbonization, thereby delaying the internal thermal pyrolysis of the load-bearing fiber strands within the web junctions. Accordingly, the scientific novelty is limited to evaluating the residual mechanical performance of these molded GFRP configurations after controlled thermal exposure. The main purpose of the study is to contribute to determining new design limits for industrial floor, platform, and staircase systems exposed to high temperatures by revealing the effect of increasing temperature on the structural safety of GFRP systems at the numerical and microscopic levels. By moving beyond baseline material-scale degradation and isolating the interplay between macro-structural profile geometry, physical resin softening (Tg), and macromolecular matrix pyrolysis, this work establishes a novel, multi-scale engineering benchmark for the safety of structural composites in high-temperature environments.

## 2. Materials and Methods

### 2.1. GFRP Grid Samples

**Figure 1:**
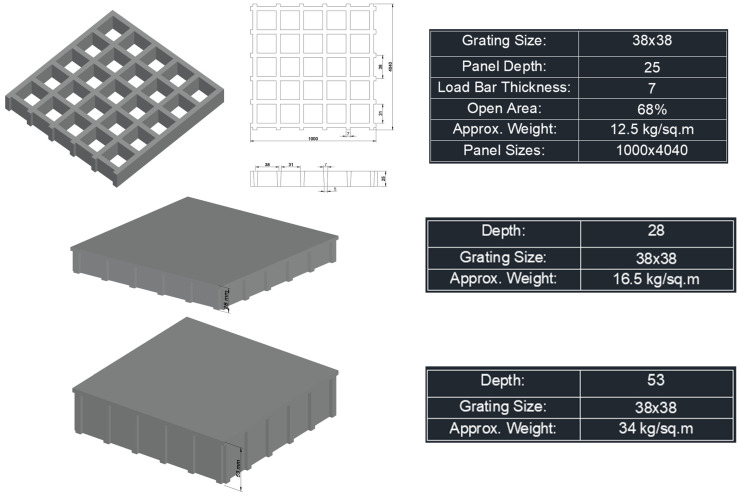
Geometric properties of GFRP Grids.

In this study, experimental investigations were conducted on GFRP grating panels industrially manufactured using the molding method. The grids used have a cell structure with square cross-sections with a nominal mesh size of 38 × 38 mm, and are systematically designed for bidirectional structural load distribution. The matrix phase of all investigated gratings consists of a premium-grade isophthalic unsaturated polyester resin that is pre-accelerated and cross-linked with styrene monomers to ensure structural integrity and elevated chemical and corrosion stability. According to the manufacturer’s data, the glass fiber ratio of the grids is in the range of 35–40%. To rigorously verify the constituent properties at the material scale, standard ignition loss (burn-off) verification tests were performed on representative control segments, confirming an average fiber weight fraction (w_f_) of 32% ± 1.5%. Accounting for the nominal densities of the E-glass fibers (2.56 g/cm^3^) and the cured polyester matrix (1.22 g/cm^3^), the calculated average fiber volume fraction (*v_f_*) corresponds to approximately 19.4%. The glass fibers used in the gratings are of the E-glass type (continuous multi-end rovings with a linear density of 2400 tex) and are integrated into the matrix with a surface treatment. The samples were prepared by cutting them to standard sizes before the experiments. The examined samples were subjected to heat treatment at different temperatures. For this process, temperature values of 80 °C, 120 °C, 200 °C, 240 °C, 280 °C and 320 °C were selected; for each temperature group, the samples were kept in an oven under isothermal conditions for 2 h. The 2-h exposure was selected to ensure comprehensive thermal equilibrium throughout the cross-section of the specimens. Given the significant thickness variations in the composite components (up to 53 mm for TCG) and the naturally low thermal conductivity of the constituent glass fibers and the polymer resin, this exposure window eliminates internal thermal lag and localized temperature gradients, ensuring that the core junctions achieve uniform, isothermal saturation. This duration is in strict alignment with established high-temperature test protocols for structural composite profiles. After heat treatment, all samples were brought to room temperature and stabilized before testing. Three different types of GFRP composite gratings were used: open grating (OG), one-surface-closed thin grating (CG), and one-surface-closed thick grating (TCG). Reflecting their distinct macro-geometric designs, the OG, CG, and TCG composites have nominal areal weights 12.5 kg/m^2^, 16.5 kg/m^2^, and 34.0 kg/m^2^, respectively. The geometric properties of the composites are given in Figure 1.

### 2.2. Temperature Applications

**Figure 2:**
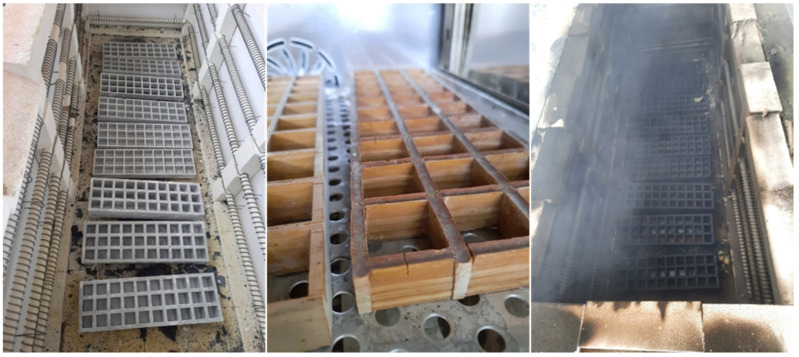
Temperature application.

Samples were acclimated to controlled temperatures before the experiment (Figure 2). For this purpose, the temperatures assigned to each test group were 80 °C, 120 °C, 200 °C, 240 °C, 280 °C, and 320 °C. A different group of samples was prepared for each temperature, and the grids were maintained at that temperature in a programmable air oven for 2 h. Following the thermal exposure period, the samples were removed from the oven and allowed to cool down to room temperature (24 ± 2 °C) via natural convection to ensure thermal stabilization before the mechanical tests. The control group, which was maintained continuously at room temperature (24 °C), also served as a reference.

### 2.3. Scanning Electron Microscope (SEM)

Scanning electron microscopy (SEM) analysis was performed to examine the microstructural properties of the composite samples and to observe morphological changes occurring with temperature. SEM imaging was performed using a high-resolution device in the BİTAM laboratory at Necmettin Erbakan University. The samples were cut before testing, and the analysis surfaces, taken from the fracture surfaces, were coated with gold. Images were taken at magnifications ranging from 1000× to 10,000× for each temperature level, and parameters such as surface roughness, resin-fiber distribution, void formation, and microcrack development were evaluated. SEM images were interpreted together with other analyses, such as FTIR and TGA, and structural deterioration observed with increasing temperature was confirmed at the microscopic level.

### 2.4. X-Ray Diffraction (XRD)

X-Ray diffraction (XRD) was used to determine the degree of crystallinity and temperature-dependent structural changes in GFRP composite samples. Analyses were performed using a diffractometer at the Necmettin Erbakan University BİTAM laboratory. Each sample was measured over the 5–80° (2θ) scanning range, with a step width of 0.02° and a scanning speed of 1°/min. The samples were ground into powder and compressed onto standard glass carriers. The obtained diffractograms were evaluated for possible crystalline peaks and for the amorphous-structure signal. The changes in crystalline area ratios, peak widths, and intensities due to temperature effects were analyzed, and the deterioration of the amorphous-crystalline balance was revealed. This method directly reflected how the internal structural order was affected by temperature and, together with mechanical and chemical analyses, provided a holistic characterization.

### 2.5. Fourier Transform Infrared Spectroscopy (FTIR)

Fourier-transform infrared spectroscopy (FTIR) was used to identify chemical changes in the composite samples as a function of temperature. Analyses were performed in the ATR (Attenuated Total Reflectance) mode over the wavenumber range 4000–600 cm^−1^. 32 scans were taken for each sample, and the resolution was determined to be 0.25 cm^−1^.

To ensure methodological consistency, the specimens used for spectroscopic characterization were systematically extracted from the baseline matrix and the fiber-core constituents of the OG-type grating. Because all three investigated grating layouts (OG, CG, and TCG) are fabricated from the same isophthalic unsaturated polyester matrix and E-glass fiber reinforcement, all cured under uniform industrial processing conditions—their chemical profiles, cross-linking configurations, and molecular thermal responses are identical at the material scale. Therefore, the baseline and post-exposure molecular properties obtained via FTIR are fully representative of the structural product group investigated in this study. Surface-treated GFRP samples were directly placed on the sample table of the FTIR device and analyzed without any preliminary chemical treatment. Using this method, the spectral changes of characteristic functional groups such as –OH, C=O, C–O–C, CH_2_, and aromatic C=C in the resin structure as a function of temperature were evaluated. Comparative analyses were performed on samples pretreated at different temperatures; bond intensities, peak shifts, and peak amplitudes were considered in the interpretation of the spectra.

### 2.6. Dynamic Mechanical Analysis (DMA)

Dynamic Mechanical Analysis (DMA) was applied to evaluate the viscoelastic behavior of GFRP composite grid samples as a function of temperature. The tests were carried out in dual cantilever mode, with a constant frequency of 1 Hz and a heating rate of 10 °C/min. The samples were prepared in accordance with the standards, were approximately 50 mm in length and 10 mm in width, and the analyses were conducted over the temperature range of 25–300 °C. Storage modulus (E′), loss modulus (E″), and tan δ (E″/E′ ratio) parameters were monitored throughout the measurement. The glass transition temperature (Tg) was determined using the peak of the tan δ curve as the reference. This method enabled analysis of the elastic and viscous behavior of the material as a function of temperature and yielded important data on the service temperatures and limits of structural behavior of GFRP composites.

### 2.7. Thermogravimetric Analysis and Differential Scanning Calorimetry (TGA–DSC)

Thermogravimetric Analysis (TGA) and Differential Scanning Calorimetry (DSC) were applied concurrently to determine the thermal stability of the composite samples. All analyses were performed using the simultaneous thermal analysis device (STA) located in the Necmettin Erbakan University BİTAM laboratory (Mougins, France). While the rate of mass loss of the sample as the temperature increased was determined by TGA, endothermic and exothermic events occurring over the same temperature range were measured as heat flux by DSC. The samples were analyzed over the temperature range 25–320 °C in a nitrogen atmosphere at a constant heating rate of 10 °C/min. The measurements were made with a sample mass of 14 mg ± 0.5 mg, and a separate test group was created for each temperature. Using this method, parameters such as the decomposition mechanisms of the polymer matrix, the approach to the glass transition temperature, and the formation of the residual phase could be observed at high resolution.

### 2.8. Three-Point Bending Tests

**Figure 3:**
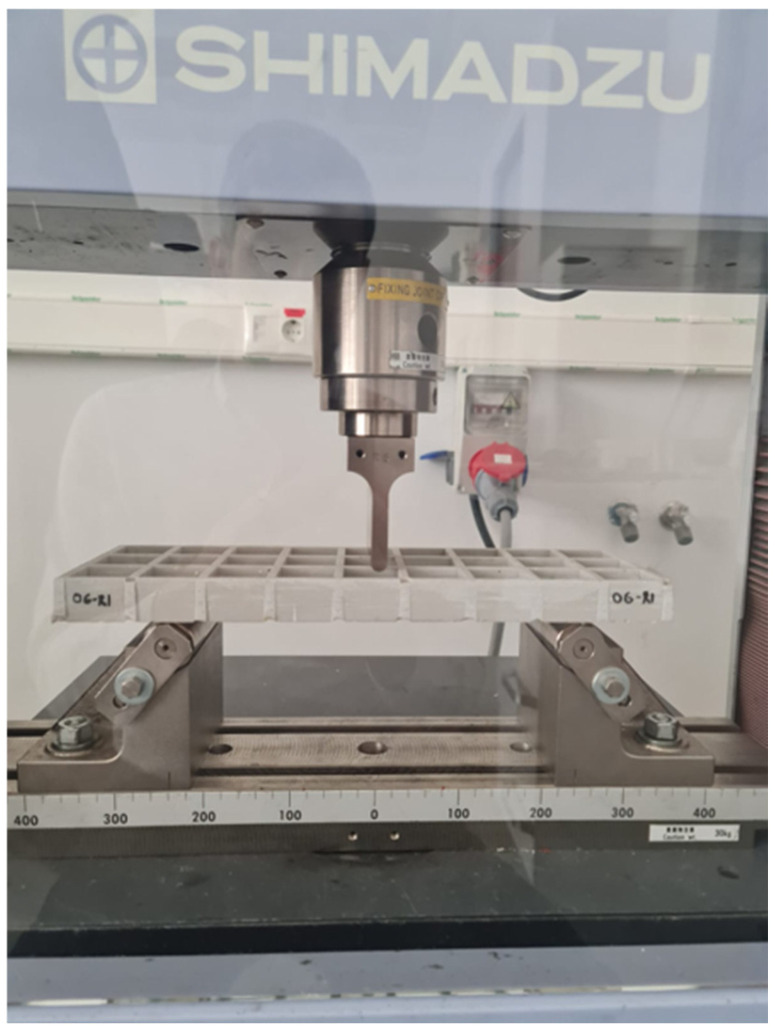
Bending test setup.

Three-point bending tests were performed to determine the mechanical load-bearing capacity of composite samples and to measure changes in structural strength as a function of temperature. The tests were carried out with a fixed support span length (L) of 240 mm. The nominal width (b) was set to 125 mm, while the nominal depths (h) of OG, CG, and TCG specimens were set to 25 mm, 28 mm, and 53 mm, respectively, providing precise baseline references for cross-sectional normalization. While the EN ISO 14125 [23] framework is traditionally intended for coupon-scale fiber-reinforced plastics, its foundational beam-theory configuration (σ = 3PL/2bh^2^) was adopted here as a volume-averaged continuum simplification. Testing full-width structural sections spanning multiple core cells was essential to capture realistic stress redistribution and global load-sharing mechanisms of the periodic grid layouts. A separate test group was created for each temperature level, and samples were tested by holding them at the relevant temperature and then returning them to room temperature. As shown in Figure 3, the experiments were carried out using an electronic SHIMADZU AGS-X (100 kN) testing machine, Shimadzu, Kyoto, Japan operating at a constant loading speed. The maximum bending loads were successfully converted into macroscopically normalized effective flexural stress (σ, MPa) and residual strength ratios (*P_T_*/*P*_0_) to explicitly isolate the geometry-independent degradation trends. In addition, parameters such as the elastic region, rupture mode, and deformation type were evaluated from the load-deformation curves. The test results clearly reveal the change in the bending strength of the GFRP structure as a function of temperature and form an integral part of other structural analyses.

## 3. Results

**Figure 4:**
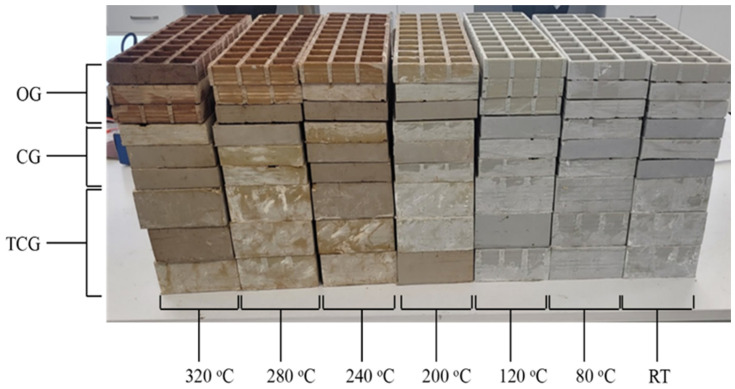
Test samples exposed to high temperatures.

Three types of GFRP gratings were exposed to elevated temperatures prior to testing. Tests were performed at temperatures of 80 °C, 120 °C, 200 °C, 240 °C, 280 °C, and 320 °C, and on a reference sample at room temperature (RT, 24 °C). The samples shown in Figure 4 are arranged from left to right in order of decreasing temperature. In other words, the visual transition from dark brown (leftmost) to light gray (rightmost) strongly correlates with an increase in the thermal resistance of the matrix (resin) system in the GFRP grating. The color change caused by temperature is clearly shown in Figure 4.

**Figure 5:**
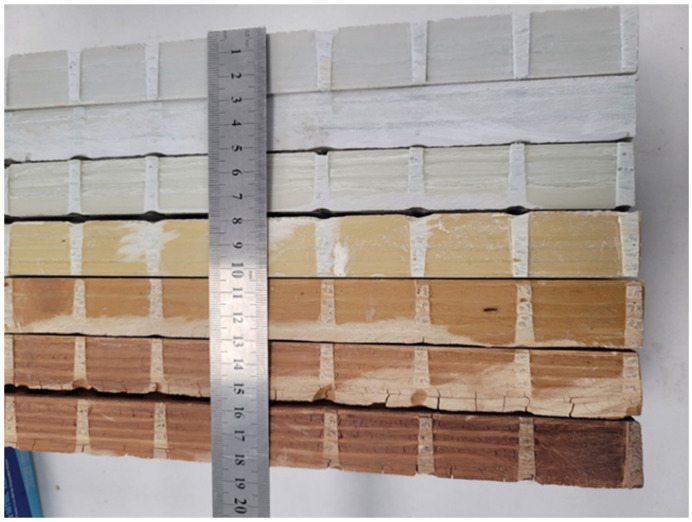
Side view of test specimens exposed to high temperature.

The side view of the samples is shown in Figure 5. The darkest-colored samples exhibit severe degradation and carbonization of the resin, which is a clear indication of resin pyrolysis (thermal decomposition). The cracks are generally concentrated at the edges of the grids. These cracks indicate the loss of the interfacial bond between the fiber and the matrix. Especially in the highest-temperature samples, pronounced cracks were observed at the grid intersections and along the longitudinal direction of the GFRP bars. Samples exposed to temperatures of 280–320 °C exhibited burnt, rough surfaces with microcracks, indicating brittleness and loss of structural integrity. In contrast, samples heated at 80 °C or 120 °C maintained smoother surfaces and a more homogeneous appearance, indicating that damage to the polymer phase was limited at these temperatures. Based on observed damage and visual inspection, flexural strength and stiffness of GFRP gratings are predicted to decrease sharply at temperatures above 200 °C due to resin embrittlement and loss of matrix continuity. Grating specimens exposed to 80 °C and 120 °C are expected to exhibit performance comparable to, or slightly lower than, the reference, likely owing to moderate softening and relaxation of the resin.

**Figure 6:**
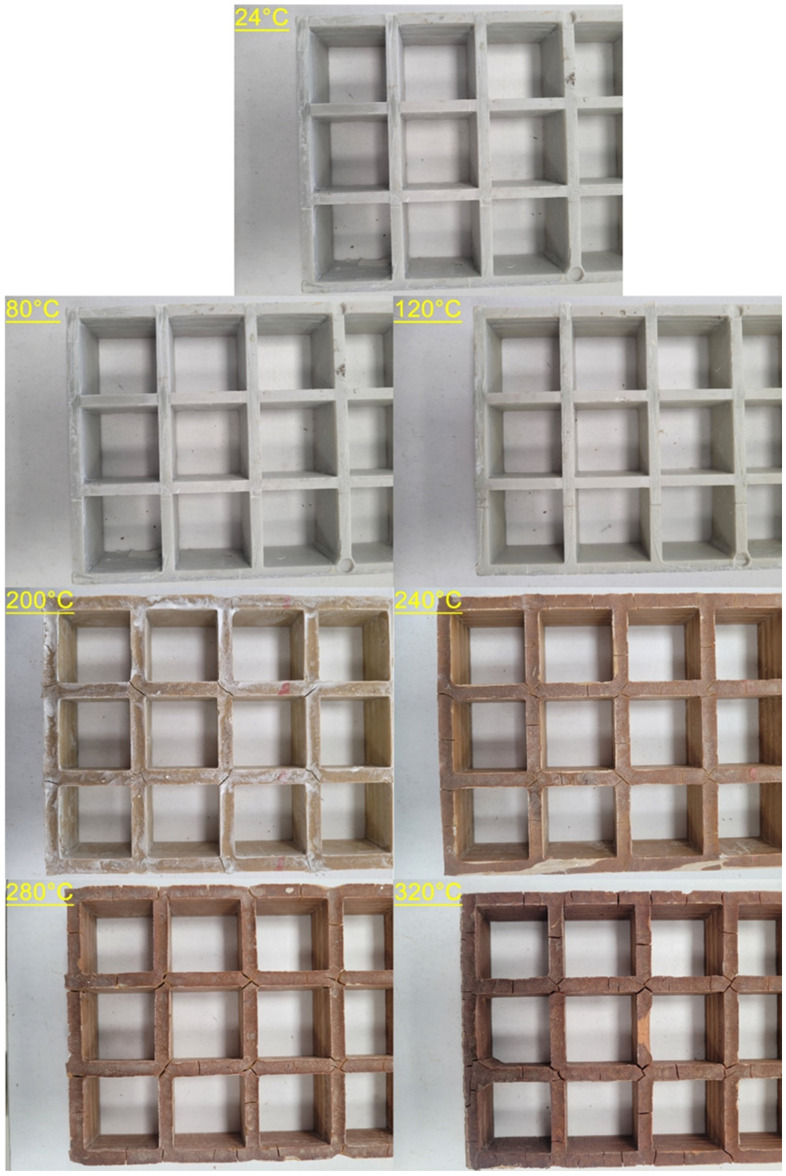
Front view of test specimens.

Figure 6 shows the front view of the samples. The control sample has a clean appearance, with a uniform grey color, sharp edges, and distinct intersections. There are no signs of surface deterioration or deformation, indicating that the polymer matrix and the fiber–matrix interface have retained their original integrity. At 120 °C, a slight color change appears, manifesting as whitening or a faint haze, which may be due to surface oxidation or thermal softening of the resin. The geometric stability is fully maintained, and the grid edges are undamaged. At 200 °C, the first signs of matrix microcracks and surface scaling appear, as well as pronounced yellowing and a chalky surface appearance. Significant resin shrinkage is observed at the intersections. At high temperatures, such as 240 °C and 280 °C, the matrix begins to brown and the resin burns, becoming more deeply discolored, especially at the intersections. As the structural matrix becomes brittle, delamination becomes more pronounced. At 320 °C, the darkest and most damaged sample shows carbonization of the resin and complete thermal collapse of the polymer matrix, with intense dark brown to black hues. Visible cracks and fractures at the nodes indicate complete embrittlement.

### 3.1. Microstructural Observations (SEM)

The changes in the microstructure of the GFRP gratings with increasing temperature were examined in detail using SEM images. The images reveal how the glass-fiber-reinforced resin changes with temperature in terms of both interfacial and matrix integrity.

The present microstructural investigation intentionally focuses on interface-scale and matrix-scale physical damage mechanisms (such as debonding, micro-cracking, and matrix disintegration) that directly govern the structural safety of the gratings. While sub-micron atomic imaging or localized elemental mapping (such as EDX) could provide micro-area atomic details, the global chemical alterations, macromolecular bond cleavage, and thermal carbonization mechanisms in the bulk composite are more comprehensively and quantitatively validated by the FTIR and TGA–DSC profiles presented in subsequent sections.

**Figure 7:**
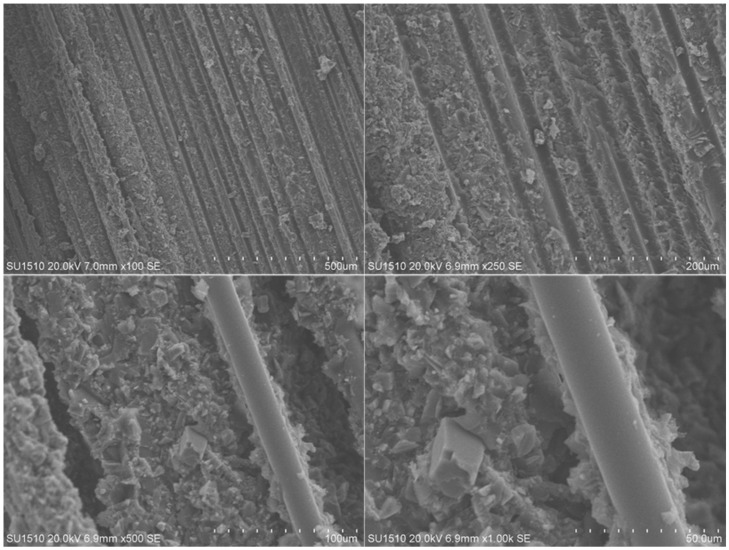
SEM images of reference sample (24 °C).

SEM images of the samples at room temperature (Figure 7) show that the initial microstructural integrity of the GFRP grids is high. The glass fibers are uniformly distributed in the resin matrix, and no separation, porosity, or crack formation is observed at the fiber–matrix interface. The fibers are regularly oriented, and a distinct fiber organization is evident throughout the structure. The resin completely surrounds the fibers and forms a compact, void-free structure, indicating that the sample has a high potential for mechanical strength. This temperature constitutes the reference level for the microstructure of the GFRP composite.

**Figure 8:**
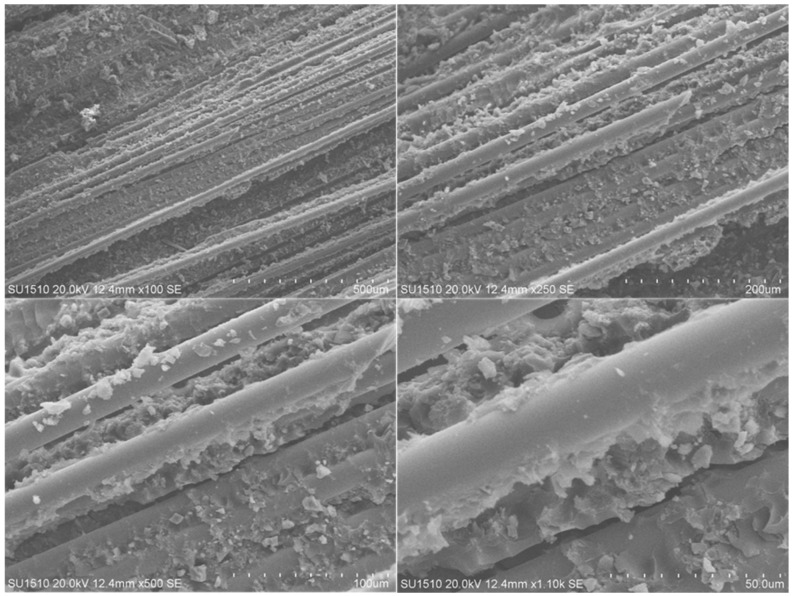
SEM images of sample (80 °C).

SEM images (Figure 8) of samples heated to 80 °C reveal that the structure has begun to deteriorate at the microscopic level. It was determined that the resin was locally withdrawn from around the fibers, and micropores formed at certain points. Weakening of the fiber–matrix bonds was observed, but the general fiber orientation and structural integrity were largely preserved. At this temperature, loosening and separation at the interface can be regarded as initial micro-deterioration caused by thermal effects. It is predicted that the structure still maintains its functional integrity at this stage, but this integrity will be more seriously damaged at higher temperature levels.

**Figure 9:**
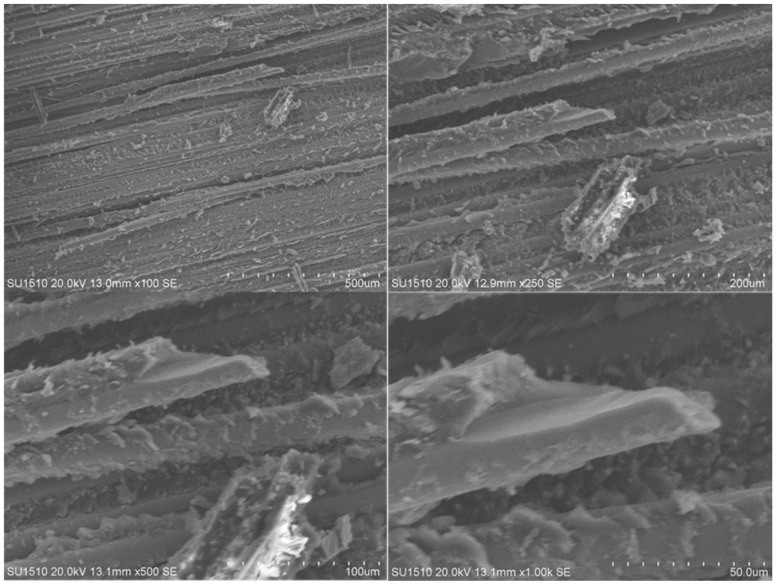
SEM images of sample (120 °C).

SEM images obtained at 120 °C (Figure 9) show that microstructural deterioration in the GFRP grid structure is apparent. The resin phase has partially withdrawn from the interface between the fibers, creating irregular boundaries and micro-voids. Crack-like separations are observed in the matrix structure around the fibers, suggesting that adhesion has weakened and the energy-absorption capacity has decreased. In some areas of the images, the fibers appear nearly separated from the resin, and microscopic traces of delamination are visible. Although the fiber orientation remains largely preserved, loss of stiffness and bond deterioration are clearly observed throughout the structure. This temperature represents a critical transition phase in which the glass transition temperature is first exceeded, indicating at the microscopic level that the thermomechanical and chemical resistance has begun to weaken.

**Figure 10:**
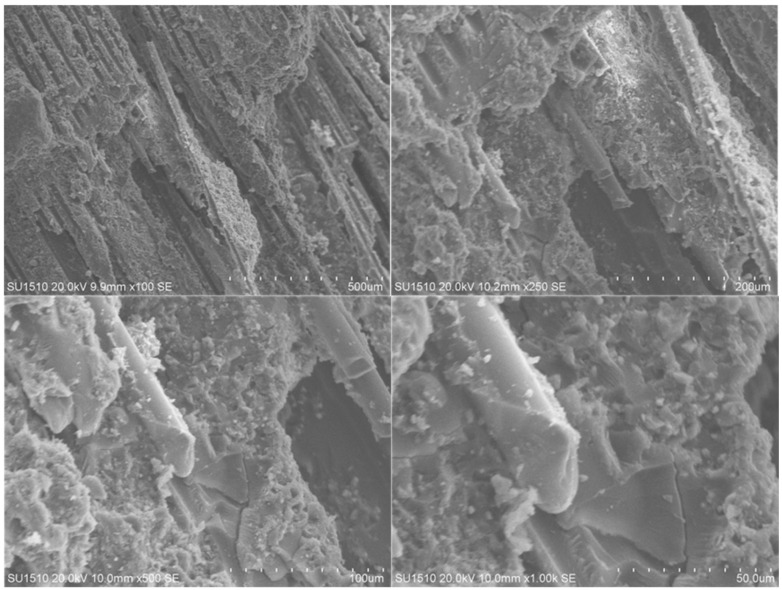
SEM images of sample (200 °C).

When the temperature reaches 200 °C, marked microstructural disintegration is observed in the GFRP grid samples (Figure 10). SEM images reveal that the resin phase around the fibers has been largely disrupted, and the interfacial contact has been lost at many points. The fibers have lost contact with the matrix and protruded onto the surface, creating large gaps around the fibers. This structural disintegration has also caused the fiber orientation to become irregular. The matrix phase has gained viscous properties, and the adhesion strength has decreased. It is also evident that in some areas, the fibers are close to breaking or not completely embedded in the matrix. This temperature marks the threshold beyond which the composite structure substantially loses mechanical and microstructural stability.

**Figure 11:**
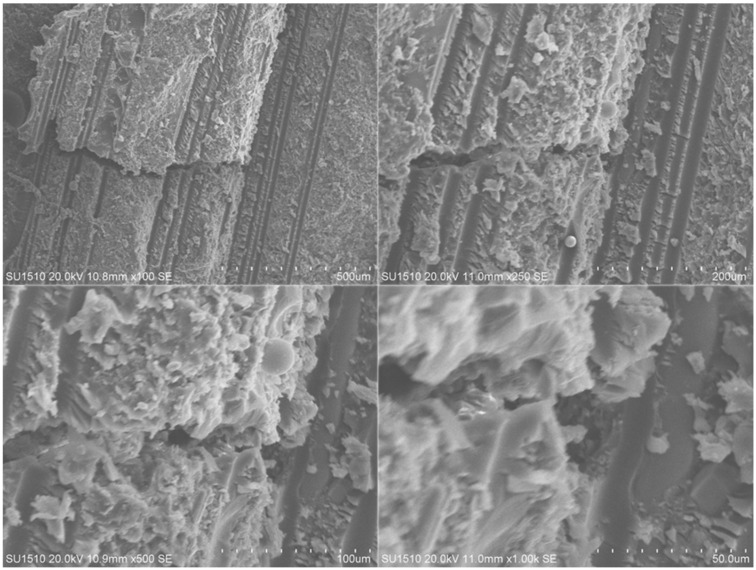
SEM images of sample (240 °C).

SEM images obtained at 240 °C (Figure 11) reveal that the modifications observed in the GFRP structure have reached a significant microstructural degradation threshold. The matrix phase around the fibers exhibits pronounced thermal damage, and the fibers are extensively exposed in many regions, with minimal resin encapsulation. The fiber–matrix interface bonds are severely compromised, and substantial micro-voids and interconnected micropores have developed in the structure. The local fiber orientation has been partially disrupted; some fibers have broken or been displaced non-directionally. This microstructural irregularity indicates that the load-bearing and energy-absorption capacities of the sample have decreased significantly, which perfectly match the quantitative strength reductions observed in Table 1. At this temperature, the resin has softened past the glass transition and transitioned to a viscous phase. 240 °C can be considered a critical thermal degradation threshold for the GFRP structure.

**Figure 12:**
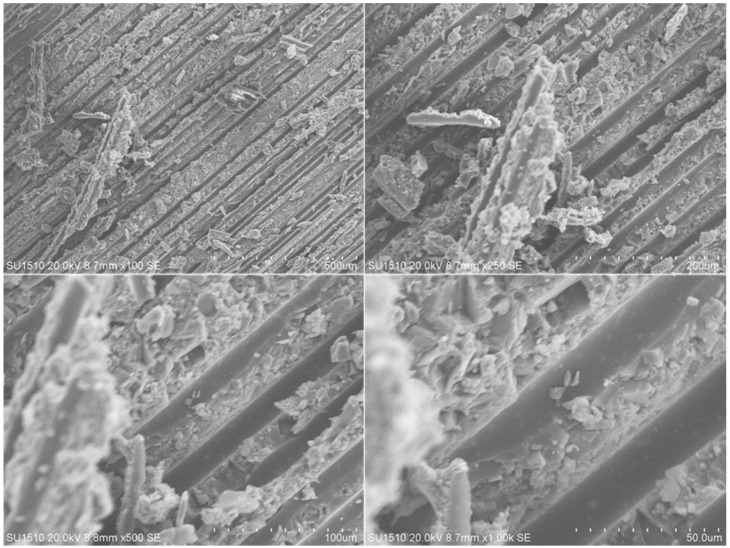
SEM images of sample (280 °C).

SEM images (Figure 12) acquired at 280 °C reveal that the GFRP structure has experienced a severe loss of its initial structural integrity. The matrix phase exhibits progressive mechanical disintegration from the fiber perimeter, losing its binding efficacy, thereby severely restricting the shear-transfer capabilities at the interface. Extensive internal void formation, local fiber clustering, and non-directional fiber orientations are clearly observed in the micrographs. This microstructural condition reflects an advanced stage of thermomechanical degradation in which mechanical load-bearing capacity is substantially reduced and deformation resistance is largely lost. This temperature range should be considered the limit at which the GFRP will be taken out of service.

**Figure 13:**
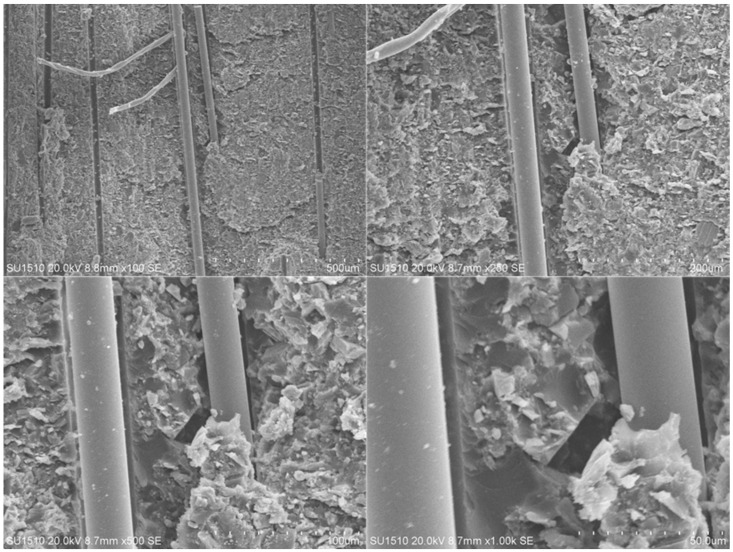
SEM images of sample (320 °C).

When the temperature reached 320 °C, the GFRP grid samples exhibited comprehensive microstructural disintegration (Figure 13). SEM images indicate that the fibers are highly irregular and essentially randomly distributed, suggesting that the isophthalic unsaturated polyester matrix has undergone severe thermal degradation and extensive pyrolysis. The cross-linked interfacial bonds are substantially reduced, and the remaining bare E-glass fiber bundles are largely isolated among extensive thermal voids. In some areas, the exposed surfaces of the individual fibers are observed to be micro-cracked or fractured. In these zones, where only highly carbonized resin residues are visible, the material is converted into an unbonded matrix-fiber skeleton. This extreme temperature level represents a threshold well beyond the serviceability limit of the GFRP material at which the cumulative microstructural damage corresponds to minimal residual mechanical properties.

### 3.2. Chemical Structure Analysis (FTIR)

**Figure 14:**
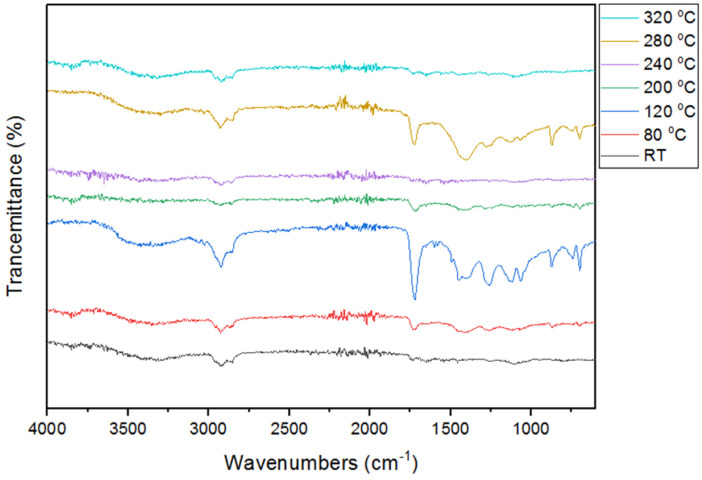
Comparative view of FTIR spectra of GFRP samples.

The FTIR spectra shown in Figure 14 were used to assess the thermal stability and chemical degradation of GFRP and calcite-filled composites exposed to isothermal conditions ranging from room temperature (RT) to 320 °C. With this method, the main functional groups in the polymer matrix, including hydroxyl (-OH), carbonyl (C=O), ether (C-O-C), methylene (CH_2_), and aromatic C=C groups, were identified, and structural changes at different temperatures were monitored.

At room temperature (RT), the FTIR spectrum exhibits well-defined, intense peaks, indicating the chemical integrity of the composite. The broad band about ~3400 cm^−1^ corresponds to -OH stretching, while the acute peak at ~1720 cm^−1^ represents ester carbonyl (C=O) bonding. Peaks in the ~1240–1150 cm^−1^ region indicate C-O-C asymmetric and symmetric stretching vibrations. Furthermore, the bands at ~2920 and 2850 cm^−1^ correlate with CH_2_ stretching, while the band at ~1600 cm^−1^ is associated with aromatic C=C vibrations. These characteristics together suggest that the polymer matrix is chemically intact and functionally active. FTIR analysis indicates that the matrix is primarily composed of a polyester with both aliphatic and aromatic groups [24]. At room temperature, Si-O-Si and Si-O-Al tensile vibrations in the range of ~1100–1000 cm^−1^ suggest the presence of glass fiber in the structure. Calcite (CaCO_3_) doping results in distinct bands at ~875 cm^−1^ (out-of-plane bending of CO_3_^2−^) and ~710 cm^−1^ (in-plane bending of CO_3_^2−^). This indicates that the mineral phase is uniformly distributed in the matrix and chemically inert.

At 80 °C, the chemical structure begins to change. The –OH band grows somewhat broader and more intense, indicating improved hydrogen bonding. The C=O and C-O-C sections show minimal alterations, indicating that the structure remains stable. However, a decrease in the intensity of the CH_2_ and aromatic C=C bands suggests the onset of segmental mobility and relaxation in polymer chains. The Si-O vibrations of the glass fiber (~1100–1000 cm^−1^) remain strong and regular at this temperature, indicating that the inorganic phase is not affected by heat. The peaks of calcite around 875 cm^−1^ and 710 cm^−1^ remain constant.

At 120 °C, considerable spectral shifts are observed, suggesting the onset of thermal transitions as the material approaches the glass transition temperature (Tg). The –OH band exhibits significant broadening and an increase in intensity, most likely due to enhanced hydrogen bonding and water loss. The strength of the C=O peak decreases, indicating that ester bonds are weakening. The C-O-C bands show broadening and spectral shifts, indicating bond rearrangement within the matrix. The Si-O peaks of the glass fiber (~1000–1100 cm^−1^) and the ~875 cm^−1^ and ~710 cm^−1^ peaks associated with calcite are visible.

At 200 °C, heat deterioration becomes noticeable. The -OH region exhibits decreased intensity and reduced peak definition, indicating that hydrogen bonding has been disrupted. The C=O peak gradually fades and undergoes a spectral shift, confirming bond cleavage. The C-O-C region becomes less defined, resulting in significant reductions in the intensity of the CH_2_ and aromatic C=C regions. These results indicate the onset of chemical degradation and physical damage within the polymer matrix. The Si-O peaks of the glass fiber (~1000–1100 cm^−1^) and the ~875 cm^−1^ and ~710 cm^−1^ peaks associated with calcite are weakened in intensity. The phenomenon is attributed to damping by the matrix, resulting from the thermally efficient transformation of the matrix structure [24,25].

At 240 °C, the FTIR spectrum shows severe degradation. The ester carbonyl peak (~1720 cm^−1^) is nearly absent; the C-O-C signals are weak and erratic. The CH_2_ and C=C bands significantly weaken, while the -OH area becomes noisy and unresolved. These spectral signatures imply a breakdown in the functional architecture and a shift to advanced chemical degradation. The degradation of the matrix at this temperature reduces the spectral visibility of the glass fiber and calcite additives [24,25].

At 280 °C, the spectra show a nearly complete breakdown of the polymeric matrix. The carbonyl peak is virtually gone, while the C-O-C regions show weak, unstructured signals. The CH_2_ and aromatic bands are barely identifiable, while the -OH band is dominated by noise. These alterations indicate the collapse of the polymer’s functional identity and the occurrence of significant chain scission. The spectral visibility of added glass-fiber and calcite is low.

At 320 °C, the FTIR spectrum confirms the complete disintegration of the composite structure. All the main functional groups have disappeared or are represented by diffuse, weak signals. The spectrum shows that the organic phase is transformed into an amorphous carbon-rich residue, losing all its distinctive features. This temperature marks the threshold for complete thermal degradation and the onset of early-stage carbonization. The glass-fiber and calcite peaks were rendered practically indistinguishable by spectral noise.

### 3.3. Thermomechanical Behavior (DMA)

**Figure 15:**
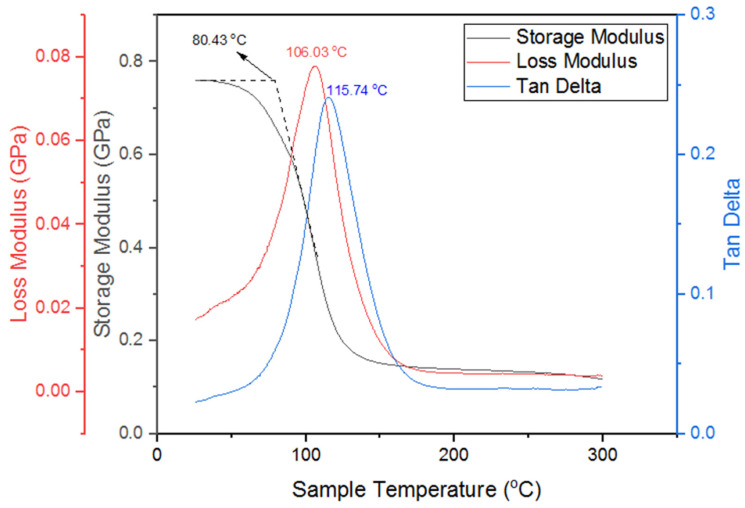
DMA curves of GFRP composite sample.

The DMA curves of the GFRP grid specimen clearly reveal significant changes in the material’s mechanical properties with increasing temperature (Figure 15). The storage modulus (E′), which is initially quite high at room temperature, decreases significantly with increasing temperature. This situation indicates that the structure is losing rigidity and its capacity for elastic deformation is decreasing. As shown on the storage modulus curve, this decline begins at 80.43 °C and accelerates markedly within the 80–120 °C region. This zone represents the purely physical glass transition region where the segmental mobility of the polymer chains increases, causing a non-destructive physical softening of the matrix rather than any chemical decomposition.

The loss modulus (E″) shows that viscous fluidity increases with increasing thermal energy, reaching a distinct peak at 106.03 °C. Subsequently, the tanδ curve reaches a maximum at 115.74 °C, which is defined as the glass transition temperature (Tg) of the cross-linked polyester matrix. Above this Tg threshold, E′ reaches a localized plateau at a lower level; however, it is critical to differentiate this physical state change from chemical degradation or from total loss of load-bearing capability. Simultaneously, the decrease in the tan δ curve indicates a reduction in energy absorption capacity. Although the matrix experiences a reduction in thermomechanical stiffness within the 80–120 °C band due to this physical phase change, macro-scale mechanical results in Table 1 confirm that the structural panels retain a substantial portion of their flexural capacity (e.g., up to a 96.9% residual strength ratio for TCG at 120 °C). This demonstrates that physical resin softening near Tg does not induce immediate chemical pyrolysis or mass loss, and that the continuous glass-fiber network maintains structural load sharing until the actual chemical decomposition thresholds, verified at much higher temperatures, are reached.

The free volume in the polymer matrix, which is the molecular-scale void space that promotes segmental chain mobility, increases when the composite material is heated above its glass transition temperature (Tg) and subsequently cooled to room temperature. This heat cycling process weakens intermolecular connections between polymer chains, leading to a decrease in overall mechanical performance. This physical rearrangement and increase in free volume compromise the filler–matrix interfacial adhesion, which correlates with the progressive, geometry-dependent residual strength regressions recorded under subsequent mechanical testing, without implying an early chemical combustion of the constituents [26].

### 3.4. Thermal Stability Analysis (TGA–DSC)

**Figure 16:**
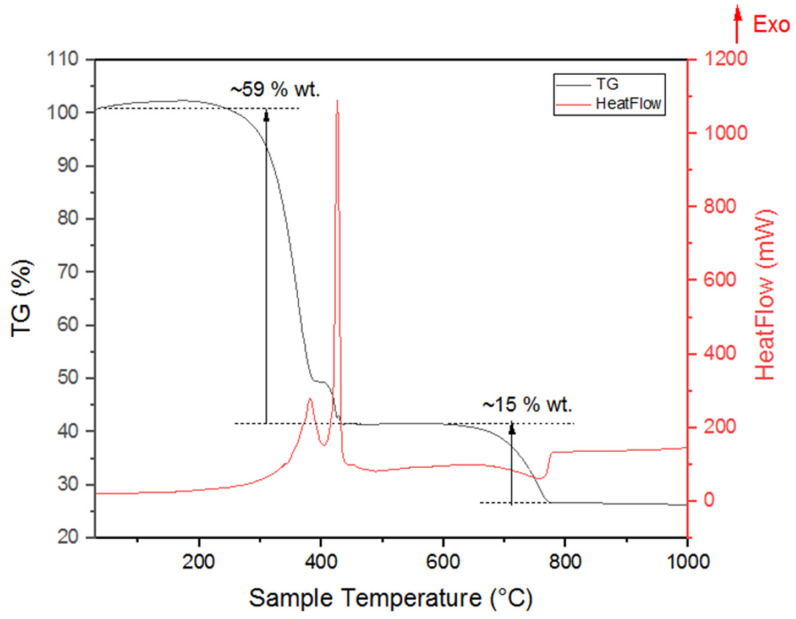
TGA–DSC curves of GRFP combustion in the oxygen atmosphere.

The GFRP composite was subjected to TGA–DSC examination in an oxygen atmosphere. The sample was heated from ambient temperature to 1000 °C at a rate of 10 °C/min. According to the thermogram curves of the composite displayed in Figure 16, the polyester matrix begins to burn at approximately 310 °C and breaks down into two separate phases by about 450 °C. A mass loss of about 59% by weight was observed up to this temperature.

The two-stage mass loss observed during combustion of the polyester matrix reflects thermal degradation of aliphatic structures in the first stage and of aromatic structures in the second. The DSC peak exhibits a greater exothermic response in the second breakdown stage than in the first. This is explained by the fact that the burning of carbon-rich aromatic rings releases more energy, which lends credence to the idea that the second mass loss is related to the breakdown of aromatic components [27].

The filler material, specifically calcite, is associated with the third mass loss observed in Figure 16. Around 700 °C, calcite (CaCO_3_) decomposes to yield calcium oxide (CaO). CO_2_ emissions are correlated with mass loss at this stage. One mol of CO_2_ is emitted per mol of CaCO_3_ broken down in this endothermic process (1:1 molar ratio). Excluding glass fibers, calcite constitutes about 34–35 wt % of the polymer matrix. Glass fibers and CaO constitute the remaining bulk of the material. The glass fiber content is estimated to be approximately 8 wt % by subtracting the calculated CaO mass from the residual mass observed in Figure 16. The uneven dispersion of the glass fibers made it impossible to accurately depict their volume fraction in the analyzed volume.

**Figure 17:**
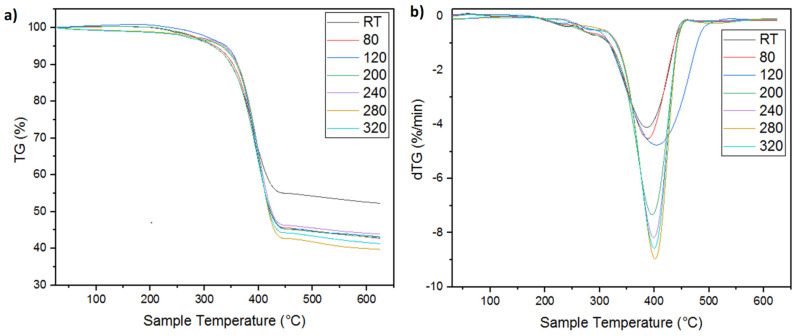
(**a**) TGA curves of GFRP samples in the nitrogen atmosphere, (**b**) dTG curves of GFRP samples.

**Figure 18:**
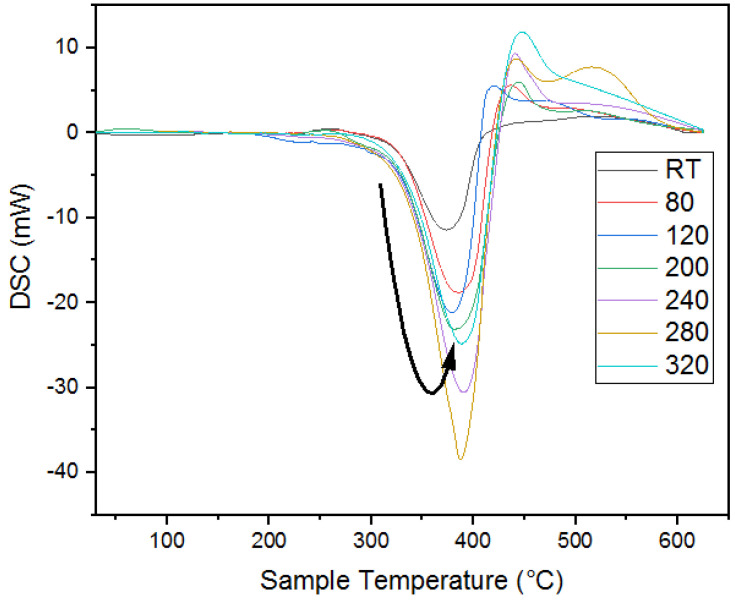
DSC curves of GFRP samples (the black arrow indicates the variation and deepening of the endothermic decomposition peak as the pre-treatment temperature increases from RT to 320 °C).

Figure 17a and Figure 18 show the TGA–DSC curves of GFRP composite samples pretreated at different temperatures in an inert nitrogen atmosphere. The samples were heated at a rate of 10 °C/min between 25 and 800 °C. After examination at room temperature, the untreated composite sample began to show signs of thermal deterioration at approximately 300–340 °C. Following heat-induced deterioration, the untreated sample’s residual mass was approximately 52% of its original weight.

The TGA curves of the thermally pre-treated samples show that their breakdown temperatures are comparable to those of the untreated sample. However, a discernible difference in residual mass attributable to the pre-treatment was observed, with the residual mass after decomposition ranging between 40 and 45 weight percent. An initial sample mass of roughly 14 mg was used for every TGA study. The detachment of end groups that have diffused into the material or formed bonds with oxygen is believed to be the primary cause of the enhanced mass loss observed after pre-treatment in the 80–280 °C temperature range [28]. Additionally, the onset of mild thermal degradation during pretreatment at this temperature likely caused the anomaly in mass loss at 320 °C. The heat pretreatments carried out at 80–280 °C increased the mobility of polymer chains, which in turn increased the reaction rate, as evidenced by the dTG curves in Figure 17b. The FTIR spectra and DMA curves shown in Figure 15 both corroborate this finding, indicating enhanced polymer chain mobility in the 80–280 °C region.

A closer look at the DSC curves in Figure 18 reveals that the pre-treated samples contain more degraded material, which further increases the energy required for decomposition. Several components partially degraded during the pre-treatment procedure, resulting in a decrease in energy absorption at 320 °C during the thermal study. This interpretation is supported by the fact that all samples exhibit an exothermic peak at temperatures above 400 °C that follows the endothermic breakdown peak. The sample pre-treated at 320 °C exhibits the strongest exothermic reaction, indicating that the oxygen that had diffused into the structure caused char formation and subsequent combustion.

### 3.5. Crystal Structure Analysis (XRD)

**Figure 19:**
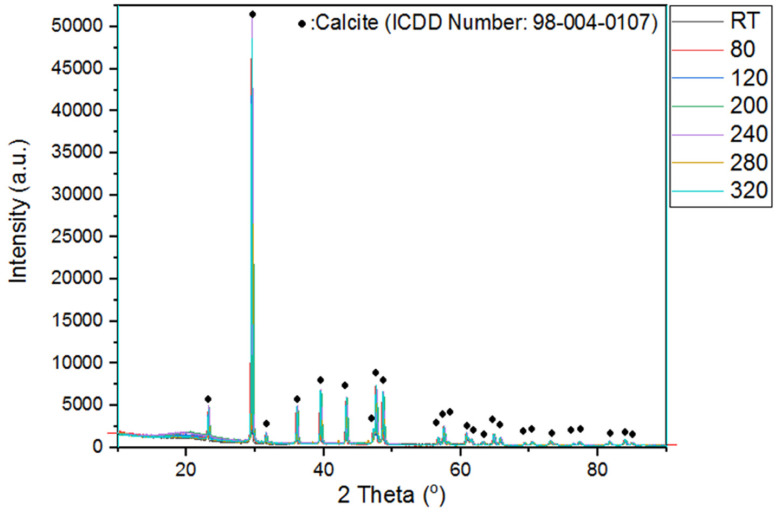
All samples XRD analysis result.

To investigate changes in the crystal structure of GFRP gratings as temperature increased, XRD analyses were performed at each temperature level. Measurements were taken over the range 2θ = 10–90°, and the intensity, width, and distribution of crystalline phases were compared as a function of temperature. XRD results are presented in Figure 19. Given the strong crystallinity of calcite in the composite structure, the peaks associated with this phase (ICDD Number: 98-004-0107) are in good agreement with those observed in the XRD spectrum in Figure 18. The broadening region between 15° and 22° represents the glass fibers and the polymer matrix, demonstrating their amorphous nature [29].

**Figure 20:**
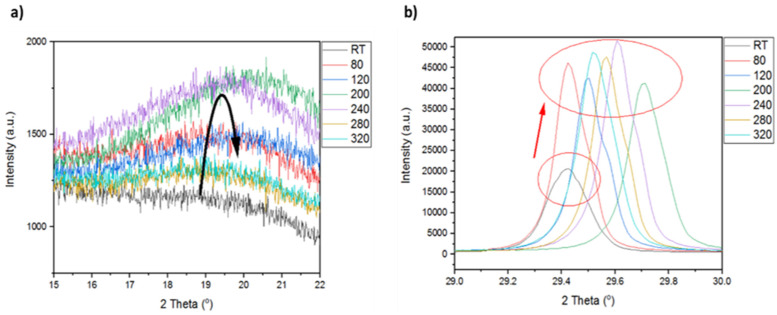
All samples XRD analysis result (**a**) humps of amorphous structure around 20° (the black arrow indicates the structural variation with temperature), (**b**) main peaks of calcite filler phase XRD pattern (the red circles and arrow highlight the peak shift toward higher 2 Theta (^0^) values and the increase in intensity with increasing temperature).

The XRD spectrum, broad between 15° and 22° and indicative of an amorphous structure, exhibits increased intensity as the pre-treatment temperature rises to 280 °C, as shown in Figure 20a. The degree of amorphous character in the structure increases because both polyester and glass fibers contribute to the amorphous region and the polymer chains are mostly broken down into smaller units up to this temperature. The amount of amorphous material, however, diminishes as the polymer matrix starts to break down at approximately 300 °C, thereby reducing the intensity of the broad peak observed between 280 °C and 320 °C.

The main peaks associated with the calcite phase are more intense in the thermally pretreated samples, as shown in Figure 20b. This observation is linked to the formation of a white residue on the samples’ surface, suggesting that thermal pre-treatment exposes calcite at the polymer-matrix surface. The peak intensities increase in direct proportion to the amount of exposed material because the calcite filler is more prominent at the surface.

### 3.6. Three-Point Bending Test

**Figure 21:**
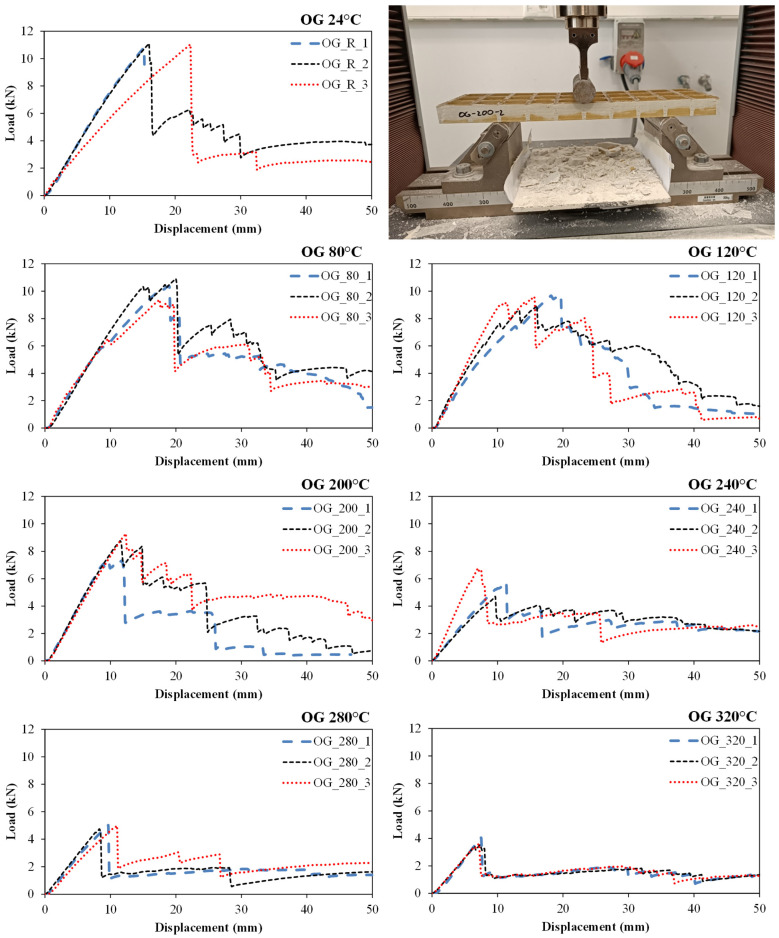
Experimental results of OG sample exposed to high temperature.

Figure 21 shows the experimental bending results of the OG specimen subjected to high temperatures. Three repetitions were performed for each specimen. The bending performance of the OG-type GFRP gratings exhibited significant deterioration with increasing temperature, as evidenced by peak load values. The average peak load at 80 °C was 10.21 kN, representing a slight decrease of 6.99% relative to the reference value of 10.97 kN at room temperature. At 120 °C, the peak load decreased to 9.39 kN, representing a loss of 14.43%. The decrease became more pronounced at 200 °C, with the average load decreasing to 8.46 kN, representing a loss of 22.95%. These results show that despite the surface softening and microcracks observed in the relevant specimens, the GFRP gratings retained a significant portion of their strength up to 200 °C. However, above 200 °C, degradation accelerated significantly.

At 240 °C, the average peak load decreased to 5.74 kN, a 47.74% reduction compared to the reference value. At 280 °C, this value decreased further to 4.91 kN, representing a 55.28% decrease. At 320 °C, the flexural strength decreased sharply to 3.72 kN, representing a total loss of 66.08% relative to the room-temperature reference. This sharp decrease in mechanical capacity at high temperatures is consistent with visual evidence such as severe resin burning, cracking of the grating intersections, and loss of interfacial bonds. These findings confirm that the structural integrity of OG-type GFRP gratings is severely compromised at temperatures above 200 °C due to progressive resin degradation and embrittlement.

**Figure 22:**
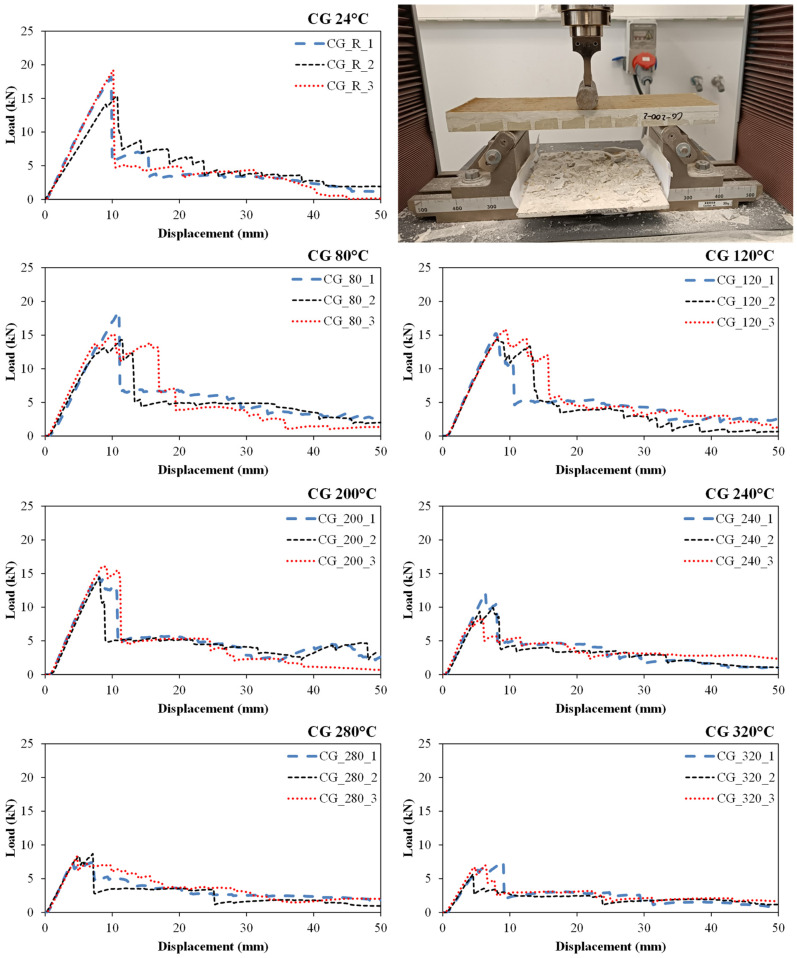
Experimental results of CG sample exposed to high temperature.

Figure 22 shows the experimental results of a CG specimen subjected to high temperatures. Three replicates were performed for each specimen. The flexural performance of CG type GFRP gratings decreased as temperature increased, but remained relatively stable up to 200 °C. The average peak load decreased from 17.55 kN at room temperature to 15.93 kN at 80 °C, indicating a 9.23% decrease. At 120 °C, the load decreased slightly to 15.15 kN, indicating a 13.71% loss. The decrease continued at 200 °C, where the peak load was 14.99 kN, representing a 14.58% reduction relative to the reference. These modest reductions indicate that CG-type gratings retain most of their load-carrying capacity at temperatures up to 200 °C, which likely reflects the durability of the resin system. However, above 200 °C, CG gratings exhibited a significant deterioration in mechanical performance. At 240 °C, the average peak load dropped sharply to 10.11 kN, representing a 42.39% decrease. This decrease accelerated further at 280 °C, where the load dropped to 8.11 kN, representing a 53.81% decrease relative to the initial level. The most serious deterioration occurred at 320 °C, where the bending load dropped to 6.72 kN, representing a 61.72% decrease compared to room temperature. These sharp losses confirm that temperatures above 200 °C weaken the polymer matrix and the fiber–matrix interface in CG-type gratings, leading to embrittlement, crack propagation, and severe loss of structural integrity.

**Figure 23:**
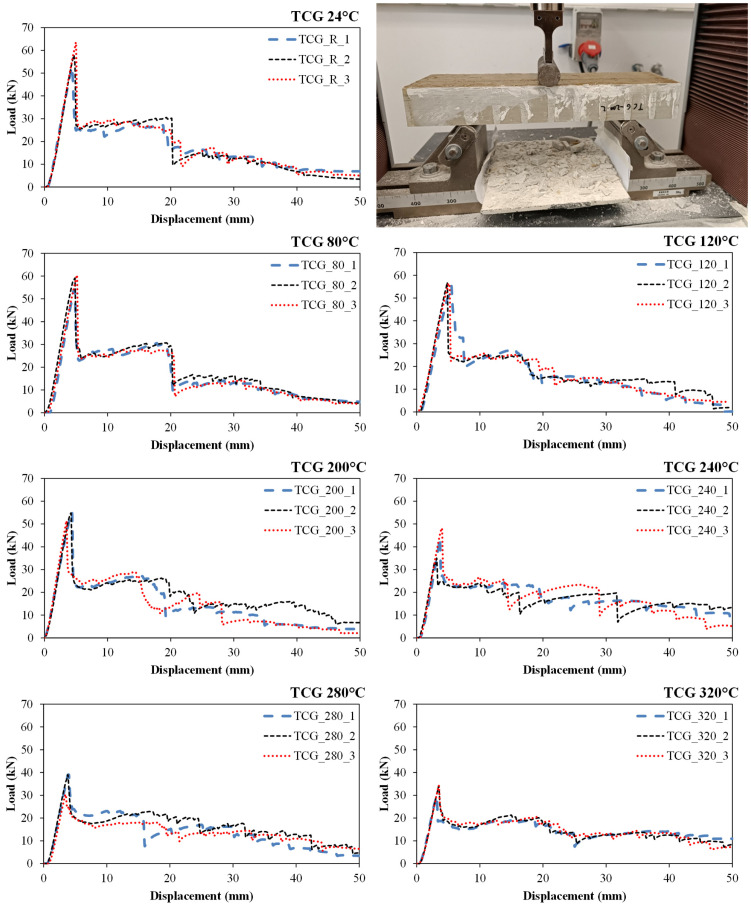
Experimental results of TCG sample exposed to high temperature.

Figure 23 shows the experimental results of TCG specimens subjected to high temperatures. Three replicates were performed for each specimen. TCG-type GFRP gratings exhibited the highest thermal resistance among the tested specimens and maintained their flexural performance at mildly elevated temperatures with minimal deterioration. The average peak load at 80 °C was 57.80 kN, showing only an insignificant decrease of 0.27% compared to 57.96 kN at room temperature. At 120 °C, the decrease was again low, 3.14%, and the average peak load was 56.14 kN. Even at 200 °C, TCG gratings retained most of their strength, with only a modest 7.32% loss and a peak load of 53.72 kN.

These results indicate that the TCG resin system exhibits excellent thermal stability and fiber–matrix compatibility up to 200 °C and is affected only to a limited extent by softening or thermal oxidation. At temperatures above 200 °C, mechanical deterioration became more pronounced. At 240 °C, the average peak load decreased to 42.14 kN, representing a 27.30% reduction. The decrease accelerated at 280 °C, where the load fell to 36.03 kN, indicating a 37.83% loss. The most severe deterioration occurred at 320 °C, where the peak load decreased to 32.52 kN, representing a total decrease of 43.90%. Despite deterioration at these high temperatures, TCG gratings outperformed OG and CG samples across the entire tested temperature range, demonstrating their superior structural strength and resistance to resin degradation and interfacial failures.

To comprehensively evaluate the mechanical degradation while neutralizing the geometric, weight, and depth variations among the OG (h = 25 mm), CG (h = 28 mm), and TCG (h = 53 mm) grating configurations, a normalization protocol based on the parameters of EN ISO 14125 [23] was implemented. The calculated normalized mechanical performance indicators, including residual flexural stress (incorporating the distinct section depth properties), residual strength ratio (*P_T_*/*P*_0_), stiffness retention, and the Coefficient of Variation (COV) representing statistical dispersion, are summarized in Table 1. As shown in the normalized data, the TCG type consistently demonstrates superior thermal resistance regardless of initial cross-sectional dimensions. At the maximum thermal exposure of 320 °C, the TCG configuration retains 56.10% of its initial room-temperature capacity (corresponding to a 43.90% strength loss), whereas the OG configuration degrades substantially, retaining only 33.90% of its initial room-temperature capacity. This behavior confirms that the thick, closed-skin barrier configuration provides a physical shielding effect, effectively retarding thermal oxidation and matrix decomposition within the core composite structure.

### 3.7. Analytical Empirical Fit

**Figure 24:**
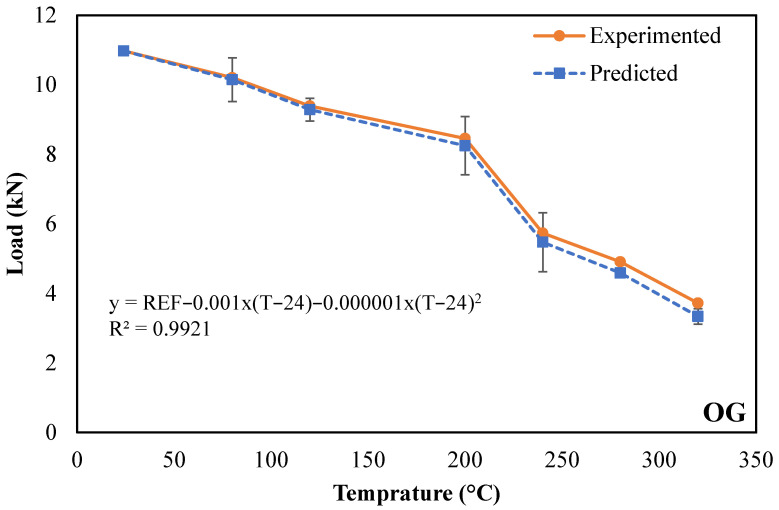
Comparison of predicted and tested results of OG samples.

The flexural performance of OG-type GFRP gratings after exposure to high temperatures was effectively predicted using the quadratic regression model. This regression framework is presented strictly as a localized empirical curve-fit (mathematical interpolation) to map the continuous, temperature-dependent trend in strength reduction, rather than as a generalized predictive or design model. The comparison between predicted and experimental results for OG specimens is presented in Figure 24. The predicted values at 80 °C and 120 °C—10.15 kN and 9.29 kN, respectively—are in very close agreement with the actual results of 10.21 kN and 9.39 kN. The deviations are less than 0.58% and 1.12%, respectively. At 200 °C, the model predicted 8.25 kN, while the experimental value was 8.46 kN, a difference of 2.45%.

In the higher temperature range, the interpolation accuracy decreased slightly due to increased material degradation. At 240 °C, the predicted load was 5.47 kN, while the actual load was 5.74 kN, representing a difference of 4.58%. At 280 °C and 320 °C, the predicted values were 4.59 kN and 3.34 kN, respectively; the actual values were 4.91 kN and 3.72 kN, corresponding to deviations of 6.55% and 10.30%.

**Figure 25:**
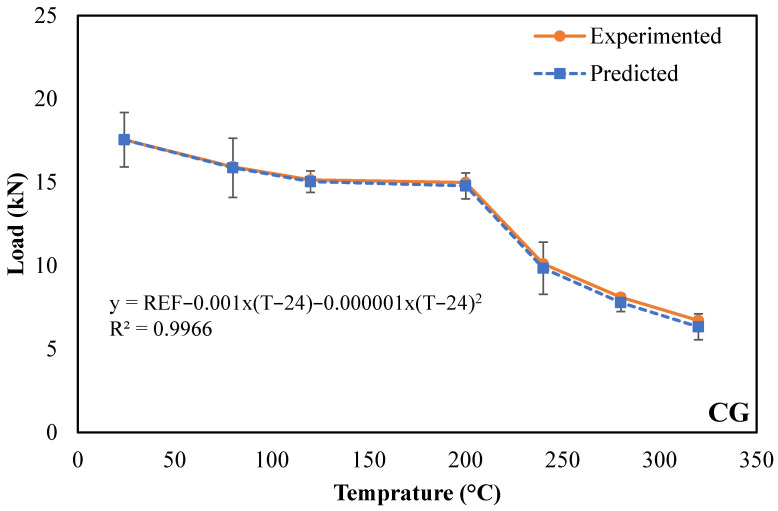
Comparison of predicted and tested results of CG examples.

The empirical fit developed for CG-type GFRP gratings accurately captures the flexural capacity at low temperatures, with deviations within practical limits. Figure 25 shows the comparison between the predicted and experimental results for CG specimens. At 80 °C and 120 °C, the model slightly underestimated the actual capacity by 0.37% and 0.69%, respectively. Even at 200 °C, when some resin degradation has already begun, the model’s predicted value is only 1.38% lower than the experimental result, indicating accurate performance up to this threshold. However, as the temperature increases further, the accuracy of the equation decreases because more severe thermal degradation mechanisms are not fully captured by the regression equation. At 240 °C, the underestimation rate increased to 2.60%, while at 280 °C and 320 °C, these rates increased to 3.97% and 5.71%, respectively.

**Figure 26:**
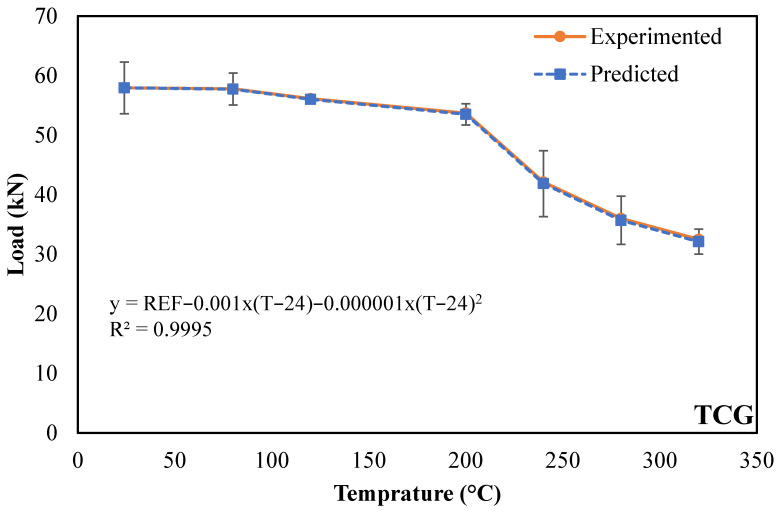
Comparison of predicted and tested results of TCG samples.

The empirical fit constants derived for TCG-type GFRP gratings exhibit excellent accuracy over the entire temperature range. Figure 26 compares predicted and experimental results for TCG specimens. The predicted values at 80 °C and 120 °C are 57.74 kN and 56.04 kN, respectively, showing insignificant differences of only 0.10% and 0.19% compared to the actual values. Although a slight thermal softening began at 200 °C, the model predicted a capacity of 53.72 kN, which is very close to 53.51 kN, resulting in a small error rate of only 0.39%. At higher temperatures, the model largely maintained its accuracy despite increased material deterioration. The predicted capacity values at 240 °C, 280 °C and 320 °C are 41.87 kN, 35.71 kN and 32.13 kN, respectively compared with the actual values of 42.14 kN, 36.03 kN and 32.52 kN, yielding error rates of only 0.62%, 0.89% and 1.18%, respectively, indicating that the model is consistently reliable. Overall, the TCG grid model shows superior performance compared with the OG and CG types in prediction stability, confirming the superior thermal durability of the TCG resin system and the model’s suitability for predicting strength even under high-temperature exposure.

These results confirm that the model is highly effective in fitting the flexural strength for temperatures up to 200 °C, although careful calibration or support from experimental data is required to maintain accuracy at higher temperatures. Overall, the model provides accurate predictions across the entire temperature range, performs particularly well below 200 °C, and maintains acceptable accuracy even under severe thermal conditions. As a precaution, the prediction equation intentionally underestimates capacity, particularly at higher temperatures.

(1) $$P_{Tx}=P_{T24}-0.001(T_{x}-24)-0.000001{\left(T_{x}-24\right)}^{2}$$

**Figure 27:**
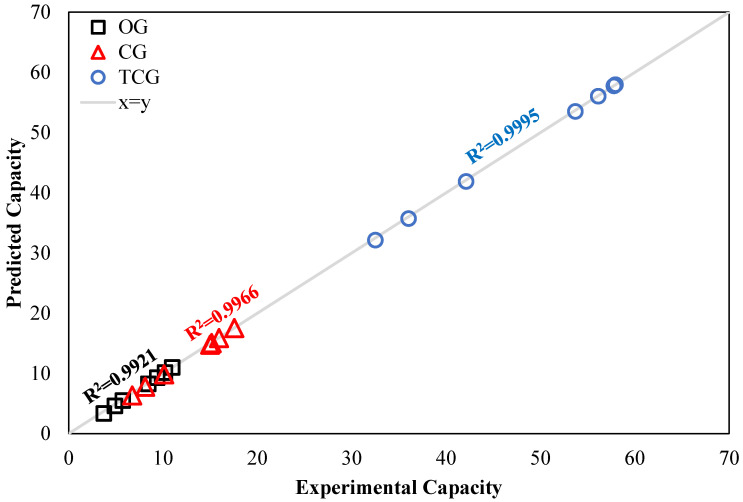
Comparison of predicted and tested results of all examples.

Equation (1) was used to estimate the high-temperature OG, CG, and TCG samples. The formulation was thoroughly checked for dimensional consistency, and the empirical constants are specific to the cross-sectional geometry, support span, and material fractions of the tested profiles. The estimated high-temperature capacity (P_Tx_) was calculated from the room-temperature capacity (PT24) and the exposed high-temperature value (T_x_). Accuracies of the estimated capacity were determined to be R^2^ = 0.9921, 0.9966, and 0.9995 for the OG, CG, and TCG grating types, respectively. Figure 27 shows a comparison of estimated and experimental capacity values for all samples.

However, a critical statistical limitation must be explicitly stated regarding the exceptionally high value (>0.99). In statistical modeling on a controlled, relatively small experimental dataset, such near-perfect correlation coefficients carry an inherent risk of overfitting. Because the model lacks validation against an independent external dataset and does not include formal confidence intervals for residuals, its mathematical applicability is strictly bounded by the evaluated temperature limits (24 °C to 320 °C). Therefore, Equation (1) should be used solely as a practical curve-fitting summary of this specific experimental program, rather than as an extrapolated, generalized structural design guideline.

## 4. Conclusions

Thermo-mechanical degradation of molded GFRP grating composites subjected to temperatures from 80 °C to 320 °C was thoroughly investigated. The main findings of the multidisciplinary experimental program involving mechanical tests and structural, chemical, thermal, and crystallographic analyses are summarized in the following bullet points:

- The thermomechanical degradation of the investigated GFRP structural gratings progresses through two distinct, decoupled stages: a low-temperature physical softening phase and a high-temperature chemical decomposition phase.
- All GFRP types exhibited significant reductions in mechanical performance at temperatures above 200 °C. In particular, resin degradation, fiber–matrix interface decomposition, and microcrack formation caused serious decreases in flexural strength. While OG-type gratings lost 66.1% of their initial strength at 320 °C. They exhibited higher residual capacity, retaining 54.3% of their baseline strength, due to their thick protective profile layout acting as a passive geometric thermal barrier.
- Dynamic Mechanical Analysis (DMA) data revealed that the physical glass transition temperature (Tg) occurs at a tan*δ* peak of 115.74 °C. The mechanical stiffness reductions observed within the 80–120 °C range are solely driven by the physical softening and increased viscoelastic mobility of the polymer chains, without causing any associated mass loss, chemical combustion, or irreversible bond cleavage.
- FTIR and XRD analyses showed that, especially at temperatures above 240 °C, the chemical bonds in the resin structure were disrupted, the transition from crystalline to amorphous structure began, and this weakened the structural stability.
- TGA–DSC analyses confirmed irreversible chemical decomposition and macromolecular, characterized by severe mass loss and heat release at temperatures between 250 °C and 300 °C, indicating that actual matrix decomposition occurs at a much higher threshold than the physical resin softening near Tg.
- The developed analytical regression equations successfully captured the temperature-dependent flexural capacity. However, due to the bounded nature of the experimental data points, the exceptionally high correlation coefficients (R^2^ > 0.99) carry an inherent risk of statistical overfitting. Therefore, the formulation is strictly bounded by the evaluated temperature limits and must be used as a localized empirical curve-fitting trendline rather than as an extrapolated, generalized structural design tool.

Ambient temperature should be considered a critical design parameter when using load-bearing GFRP systems in structural engineering applications. The findings of this study reveal that while physical matrix softening begins to affect mechanical stiffness above 120 °C, actual permanent loss of mechanical integrity and structural disintegration due to chemical resin pyrolysis occur at higher exposure limits exceeding 240 °C. Although the present empirical models offer an accurate assessment based on short-term laboratory three-point bending tests, full commercial implementation into structural load-carrying tables should consider long-term environmental factors, creep, and multi-axial fatigue behavior. It is recommended that design tables and safety factors be recalibrated to take into account the short-term temperature effect. To broaden the industrial readiness of molded composite systems, future research should systematically focus on enhancing fire resistance by investigating the inclusion of non-halogenated flame retardants, intumescent, multi-scale active treatments, and post-fire residual load-bearing metrics following active flame exposure. Furthermore, evaluating the long-term environmental durability and synergistic degradation kinetics under simultaneous sustained loading (creep), hydrothermal aging, and cyclic thermal fluctuations remains a vital frontier for confidently finalizing comprehensive safety factors in industrial infrastructure design guidelines.

## Figures and Tables

**Table 1:** Normalized residual mechanical properties and statistical dispersion of OG, CG, and TCG molded GFRP gratings.

Grating Type	Temperature (°C)	Average Residual Peak Load *P_T_* (kN)	Calculated Flexural Stress *σ* (MPa) *	Residual Strength Ratio (*P_T_*/*P*_0_)	Flexural Stiffness Retention (%)	COV (%)
OG (h = 25 mm)	RT (24)	10.97	50.55	1.000	100.0	4.1
	80	10.21	47.05	0.931	93.5	5.2
	120	9.39	43.27	0.856	82.1	5.8
	200	8.46	38.99	0.771	74.6	6.4
	240	5.74	26.46	0.523	51.0	7.9
	280	4.91	22.63	0.448	43.2	8.5
	320	3.72	17.15	0.339	28.7	9.8
CG (h = 28 mm)	RT (24)	17.55	64.47	1.000	100.0	3.6
	80	15.93	58.52	0.908	91.2	4.4
	120	15.15	55.65	0.863	85.6	4.9
	200	14.99	55.06	0.854	83.1	5.2
	240	10.11	37.14	0.576	55.4	7.1
	280	8.11	29.80	0.462	44.8	8.0
	320	6.72	24.69	0.383	35.2	9.1
TCG (h = 53 mm)	RT (24)	57.96	59.42	1.000	100.0	2.8
	80	57.80	59.26	0.997	99.4	3.1
	120	56.14	57.56	0.969	95.8	3.5
	200	53.72	55.07	0.927	91.0	4.0
	240	42.14	43.20	0.727	71.2	5.7
	280	36.03	36.94	0.622	60.5	6.3
	320	32.52	33.34	0.561	54.3	7.2

* Note: Flexural stress values are scaled based on nominal cross-sectional references (σ = 3PL/2bh^2^) to demonstrate the normalized mechanical degradation trend (L = 240 mm, b = 125 mm, h = 25/28/53 mm).

## Data Availability

The original contributions presented in this study are included in the article. Further inquiries can be directed to the corresponding authors.
